# P01-14 National policy response to the Sustainable Development Goals: a physical activity case study of Wales

**DOI:** 10.1093/eurpub/ckac095.014

**Published:** 2022-08-29

**Authors:** Catherine Sharp, Kelly Mackintosh, Rhi Willmot, Rachel Hughes, Melitta McNarry, Karen Milton

**Affiliations:** 1 Welsh Institute of Physical Activity, Health and Sport, Swansea University, Swansea, United Kingdom; 2 Applied Sports, Technology, Exercise and Medicine Research Centre, Swansea University, Swansea, United Kingdom; 3 Dotiau Ltd, Dotiau Ltd, Monmouthshire, United Kingdom; 4 Faculty of Social and Life Sciences, Wrexham Glyndwr University, Wrexham, United Kingdom; 5 Norwich Medical School, University of East Anglia, Norwich, United Kingdom

**Keywords:** Collaboration, Legislation, Wales, Policy audit, SDGs

## Abstract

**Issue:**

Population level changes in physical activity (PA) may benefit from policy intervention. In response to the UN Sustainable Development Goals, Wales introduced legislation (the Well-being of Future Generations (Wales) Act 2015) to holistically improve health and well-being, including the translation of national policy into practice. This audit provides a case study approach that could be replicated by researchers in other countries to appraise the role of PA actions in national and sub-national policies.

**Description:**

An audit of policies published by national and sub-national public bodies between 2015 and 2020 was conducted. The list of identified policies was reviewed by an external panel to act as a ?critical friend? to verify its inclusiveness. Content of the policies were extracted and synthesised to determine: (i) how many policies included a PA action; (ii) what the drivers of those policies were; (iii) the content of the PA actions; and (iv) how the PA actions aligned with the Well-being of Future Generations (Wales) Act 2015.

**Results:**

A final list of 73 policies was obtained. Only 16 national-level documents had a PA action, which had been published by 4/13 public bodies (who are bound by the Act). Of the 19 sub-national well-being policies, 15 included PA actions. Most policies were considered reactive and varied in terms of the clarity and specificity of the actions, the assignment roles/responsibilities, and the setting of targets; all overarching principles which can be used to strengthen national and sub-national policy in the future. The most common theme of action across the national-level documents was the broad action of PA promotion, which was identified in nine of the 16 documents. The actions within the national-level documents were reflected in the sub-national well-being plans.

**Lessons:**

This research used a novel approach to assess alignment of policies related to PA and the Sustainable Development Goals (SDGs) in Wales. It provides an overview of the current status of policy related to PA in Wales, which can be used in manifestos and frameworks to shape the subsequent actions of public bodies.

**Main messages:**

This case study provides a valuable example of how to utilise PA to address broader health and wellbeing agendas, and specifically the SDGs. It also demonstrates an approach to achieving stronger connections between national and sub-national policy to support the translation of policies into practice.

